# Different responses to mechanical injury in neonatal and adult ovine articular cartilage

**DOI:** 10.1186/1475-925X-12-53

**Published:** 2013-06-17

**Authors:** Xuhong Xue, Qixin Zheng, Hongbin Wu, Lixue Zou, Peng Li

**Affiliations:** 1 Department of Orthopedics, Union Hospital, Tongji Medical College, Huazhong University of Science and Technology, Wuhan, 430022, China

**Keywords:** Cartilage, Microarray, Mechanical injury, Neonatal, Ovine, Differential gene expression

## Abstract

**Background:**

Articular cartilage injury remains a major challenge in orthopedic surgery. This study aimed to identify differences in gene expression and molecular responses between neonatal and adult articular cartilage during the healing of an injury.

**Methods:**

An established *in vitro* model was used to compare the transcriptional response to cartilage injury in neonatal and adult sheep by microarray analysis of gene expression. Total RNA was isolated from tissue samples, linearly amplified, and 15,208 ovine probes were applied to cDNA microarray. Validation for selected genes was obtained by real-time quantitative polymerase chain reaction (RT-qPCR).

**Results:**

We found 1,075 (11.6%) differentially expressed probe sets in adult injured cartilage relative to normal cartilage. A total of 1,016 (11.0%) probe sets were differentially expressed in neonatal injured cartilage relative to normal cartilage. A total of 1,492 (16.1%) probe sets were differentially expressed in adult normal cartilage relative to neonatal normal cartilage. A total of 1,411 (15.3%) probe sets were differentially expressed in adult injured cartilage relative to neonatal injured cartilage. Significant functional clusters included genes associated with wound healing, articular protection, inflammation, and energy metabolism. Selected genes (PPARG, LDH, TOM, HIF1A, SMAD7, and NF-κB) were also found and validated by RT-qPCR.

**Conclusions:**

There are significant differences in gene expression between neonatal and adult ovine articular cartilage following acute injury. They are partly due to intrinsic differences in the process of development, and partly to different biological responses to mechanical trauma between neonatal and adult articular cartilage.

## Background

Articular cartilage injury remains a major challenge in orthopedic surgery. This may be mainly due to the specific morphological structure of articular cartilage [1]. Articular cartilage is a highly ordered, specialized connective tissue, which provides a smooth surface and low friction weight-bearing support used for protection of joints by absorbing mechanical stresses and loads [2]. Traumatic cartilage injury leads to an irreversible cartilage loss because differentiated chondrocytes do not divide, and therefore, do not compensate for these defects. Previous studies have reported that post-traumatic articular cartilage in adults is often fibrous cartilage or hyaline-like cartilage of which the biological properties and mechanical strength are inferior to normal cartilage [3]. However, the results from a clinical study indicated that acute full-thickness joint surface defects show the potential for intrinsic repair in young individuals [4]. Similarly, spontaneous repair of relatively small, experimental, full-thickness joint surface defects in animal models has been reported [5]. Spontaneous repair can be complete in a fetal lamb articular cartilage superficial defects model [6].

The different mechanisms of cartilage repair in young and adult articular cartilage are unclear. Changes at the molecular level, consisting of key genes or signaling pathways, may occur during the developmental process, and this might lessen the repair ability of articular cartilage.

This study compared the transcriptional response to cartilage injury in neonatal and adult sheep. This study aimed to identify the portion of gene regulation associated (and perhaps responsible for) successful healing. Our findings could be important for designing instruments to induce cartilage repair.

## Methods

### *Ex vivo* cartilage injury model and tissue culture

Articular cartilage explants were harvested from adult (n = 3, 2 years old) and neonatal sheep (n = 3, 1 week old) bilateral femoral medial condyle. These animals were housed in the animal center of the Tongji Medical College, Huazhong University of Science and Technology. The study was approved by the Ethical Committee for Animal Experiments of Tongji Medical College, Huazhong University of Science and Technology.

**Figure 1:**
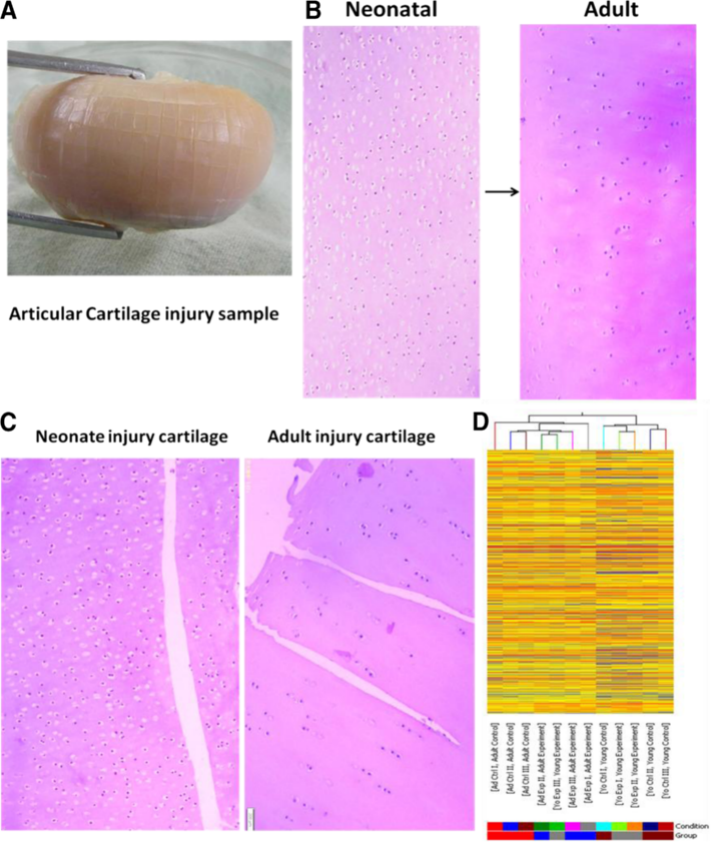
**The morphological assessment of injury/normal tissue and hierarchical clustering analysis of genes expression. A**. The model of articular cartilage injury. Articular cartilage explants were dissected onto a 2 × 2 mm^2^ grid (horizontal and vertical at 2-mm intervals). **B**. Histomorphometric comparison of isotropic articular cartilage structure in the ovine neonate and anisotropic structure in adults. **C**. Histomorphometric comparison of injured neonatal and adult articular cartilage. **D**. The resulting gene trees were grouped (samples/conditions) together based on the similarity of their expression profiles. The dendrogram shows the relationships among the expression levels of conditions.

The experimental design of cartilage injury was as follows: adult experiment (injury) versus adult control (normal); neonatal experiment (injury) versus neonatal control (normal); adult experiment (injury) versus neonatal experiment (injury); and adult control (normal) versus neonatal control (normal). Cartilage explants were washed in phosphate-buffered saline and maintained in a culture medium as previously described [7], containing Dulbecco’s modified Eagle’s medium /F12 (Invitrogen) in the presence of 10% fetal bovine serum (Invitrogen), and 100 units/ml penicillin and streptomycin (Invitrogen) in a six-well culture plate at 37°C in a humidified 5% CO_2_ atmosphere. The medium was changed every other day, and after 6 days, the medium was removed. Our model of cartilage injury is summarized in Figure 1A. Cartilage explants at left side were dissected onto a 2 × 2 mm^2^ grid (horizontal and vertical at 2-mm intervals) using a scalpel. Care was taken to avoid contamination by blood, bone, or synovium. The explant at right side was used for control samples. After 24 h, articular cartilage explants were shaved from the joint surfaces and preserved in liquid nitrogen for later RNA extraction.

### Histology

Samples were also collected and prepared for histological analyses as described by Frisbie et al. [8]. Briefly, normal articular cartilage tissue and injury were fixed in 10% neutral buffered formalin for a minimum of 2 days. Samples then had 0.1% EDTA/3% HCl decalcification solution added, which was replenished every 3 days until specimens were decalcified. Specimens were embedded in paraffin and sectioned at 5 μm. Sections were stained with hematoxylin and eosin.

### Total RNA extraction

Total RNA was isolated as described by Dell’Accio et al. [7]. Briefly, each frozen explant was pulverized using a mortar and pestle pre-chilled in liquid nitrogen, suspended in 4 ml of TRIzol reagent (Invitrogen), and homogenized using a Mini-Bead-Beater-16 (Biospec). This was followed by differential alcohol and salt precipitations, and then final purification was performed using the Qiagen RNeasy Mini Kit by following the manufacturer’s protocol. RNA quantification and quality assurance were tested by NanoDrop-1000. Purity and integrity were assessed using the Agilent 2100 Bioanalyzer. The RNA quality was selected for microarray analysis of gene expression and quantitative real-time polymerase chain reaction (RT-qPCR).

### Microarray analysis

Total RNA from each tissue sample was amplified and labeled using the Agilent Quick Amp labeling kit, and hybridized with the Agilent whole genome oligo microarray in Agilents SureHyb hybridization chambers [9]. After hybridization and washing, the processed slides were scanned with a DNA microarray scanner (Agilent, part number G2505B) using settings recommended by Agilent Technologies. Feature Extraction software (version 10.5.1.1) was used to assess fluorescent hybridization signals and to normalize signals using linear regression and a Lowess curve-fit technique. Reproducibility and reliability of each single microarray were assessed using quality control report data (Feature Extraction software, version 10.5.1.1).

### Quantitative real-time RT-qPCR

Quantitative real-time RT-PCR was performed as described previously [7]. Gene expression was calculated using a standard curve and was normalized to the expression of the housekeeping gene glyceraldehyde-3-phosphate dehydrogenase (GAPDH). Purified RNA was reversely transcribed into cDNA using Superscript II RT (Invitrogen). Equivalent amounts as calculated by the initial RNA quantity were added to the reaction mix including 12.5 ml SYBR Green (Invitrogen), forward and reverse primers (10 pmol/ml), with 0.5 ml for each primer, and nuclease-free water to final volumes of 25 ml per well. Primer sequences are listed in Table 1. Real-time RT-PCR was run in an ABI Prism 7700 Sequence Detection System (SDS) using the ABI Prism 7700 SDS software version 1.2.3.

**Table 1:** Primer nucleotide sequences used in quantitative real-time RT-qPCR assays for genes described in the study

**Gene name**	**Gene symbol**	**Primer sequences**	**Ampliconsize (bp)**
glyceraldehyde-3-phosphate dehydrogenase	GAPDH	F:5*'*GTTCCACGGCACAGTCAAGG3*'*	117
		R:5*'*TACTCAGCACCAGCATCACCC3*'*	
mothers against DPP (Drosophila)human homologue 7	SMAD7	F:5*'*ACAACCGCAGCAGTTACCC3*'*	129
		R:5*'*TGTACGCCTTCTCGTAGTCAA3*'*	
peroxisome proliferator-activated receptor gamma	PPARG	F:5*'*GCGACATCGACCAACTGAAC3*'*	274
		R:5*'*ACGGAGCGAAACTGACACC3*'*	
thappin ovine molecule	TOM	F:5*'*CCAGGTGGTGGTGCTTCTC3*'*	127
		R:5*'*ACCGTTGATTGGACCCTTT3*'*	
nuclear factor-kappa B	NFκB	F:5*'*ACGAGGATGATGAGAATGGATG3*'*	135
		R:5*'*GCAGGAACACGGTTACAGGAC3*'*	
lactate dehydrogenase	LDHA	F:5*'*GGGACAGAATGGAATCTCAGAC3*'*	296
		R:5*'*TTGCCATCCAGCAGGGT3*'*	
Hypoxia-inducible factor-1α	HIF1α	F;5*'*-CGAAGAACTCTCAGCCACAG-3*'*	174
		R:5*'*-AGCTCGTGTCCT CAGATTCC-3*'*	

### Statistical analysis

The 12 microarray data sets were normalized in GeneSpring GX (version 11.0) using the Agilent FE (version 10.5.1.1) one-color scenario (quantile normalization). The entities were filtered based on their flag values of P (present), M (marginal), and A (absent). Only entities having the present and marginal flags in at least one sample are displayed in the profile plot.

Only genes with values exceeding background intensity in at least three samples of either condition for each comparison were used for two-way analysis of variance (ANOVA) with the least significant difference (LSD) t-test, which were followed by Benjamini and Hochberg correction based on a false discovery rate of 2.2% for probe sets with a *p*-value <0.01 [10]. Volcano plots were used to filter for genes differentially expressed by ≥2-fold and with *p* < 0.05. Unsupervised hierarchical clustering analysis was performed on this subset of genes.

For quantitative real-time RT-PCR, the gene expression ratio between every two groups was determined and analyzed using SPSS version 17.0 (SPSS Inc., Chicago, IL, USA).. The relative expression levels in every two compares for the selected genes were normalized to the endogenous reference gene GAPDH by using the formula 2^-Ct target^/2^-Ct GAPDH^, where Ct is the threshold cycle. All data are expressed as mean ± standard deviation. Differences were considered significant at *p* < 0.05.

## Results

### Articular cartilage histology

Tissue samples were harvested 24 h after injury induction of full-thickness cartilage lesions. Gross histomorphometric examination showed the transition from isotropic to anisotropic architecture in neonatal and adult ovine articular cartilage (Figure 1B). Histologically, lesion tissue generally had a homogeneous matrix architecture with elongated, flattened cells that interfaced with surrounding articular cartilage. Each lesion was dimpled in appearance and not completely level with the articular surface (Figure 1C).

### Overall level of differential gene expression and annotated genes

**Figure 2:**
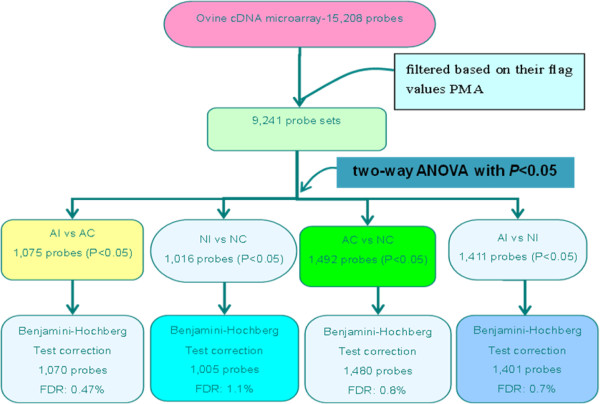
**Flowchart of cDNA microarray data analysis.** The groups are as follows: adult injury (AI), adult control (AC), neonatal injury (NI), and neonatal control (NC). Expression data were initially analyzed by PMA and two-way ANOVA with the LSD t-test, with Benjamini and Hochberg correction. A total of 11.6, 10.9, 16.0, and 15.2% of the probe sets on the microarray showed significant differential gene expression in each pair (*p* < 0.05).

Of the 15,208 gene probes, 9,252 probe sets were present in the PMA. Further analyses were carried out on these probe sets. Based on a *p*-value of 0.05, 1,075 (11.6%) probe sets were differentially expressed in adult injured cartilage relative to normal cartilage, 1,016 (11.0%) probe sets were differentially expressed in neonatal injured cartilage relative to normal cartilage, 1,492 (16.1%) probe sets were differentially expressed in adult normal cartilage relative to neonatal normal cartilage, and 1,411 (15.3%) probe sets were differentially expressed in adult injured cartilage relative to neonatal injured cartilage in each pair of samples (Figure 2).

**Figure 3:**
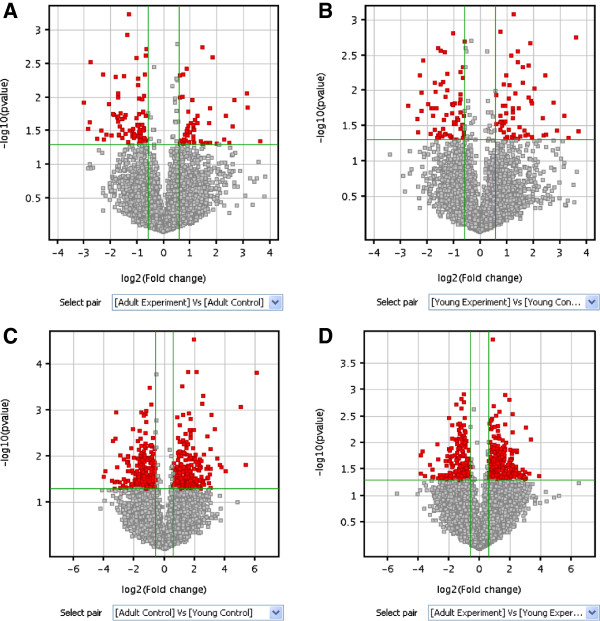
**Volcano plots of adult experiment versus control (A), neonatal experiment versus control (B), adult versus neonatal control (C), and adult versus neonatal experiment (D).** The vertical lines correspond to 2.0-fold up regulation and down regulation and the horizontal line represents a *p*-value of 0.05. Therefore, the red point in the plot represents the differentially expressed genes with statistical significance. The degree of statistical significance is displayed along the vertical axis and fold change expression is displayed along the horizontal axis.

After Benjamini and Hochberg correction to compare gene expression in the four groups, 1,070, 1,005, 1,082, and 1,401 probes were identified as being significantly (*p* < 0.05) altered in each group. The estimated false discovery rate was 0.47, 1.1, 0.8, and 0.7%, respectively (Figure 2). A volcano plot shows that 86 and 83 genes were significantly regulated at least 2-fold post-injury for neonatal sheep (Figure 3B) and adult sheep, respectively (Figure 3A). A total of 132 probe sets were up-regulated (Figure 3D) in neonatal injured articular cartilage relative to adult articular cartilage. A total of 185 probe sets were up-regulated in adult injured articular cartilage relative to neonatal articular cartilage (Figure 3D). Comparative transcription profiling and gene annotation in each pair are listed in Table 2.

**Table 2:** Comparative transcription profiling between the every two groups

**Group**	**AI/AC**	**NI/NC**	**AC/NC**	**AI/NI**
up-regulation	**32**	**190**	**185**	**44**
Annotated genes	CENP-C,LDHA,TNC,	ESR1, NF-κB, OVAR,	FZD3, NFkB1A, NOD2	SMAD7, TF, PPARG,
	DCN,TNFα,IL-1β	PRKAR1A,PBR, EF-1,	MMP7, CAT-1, RAC-1	ERBA BETA, GRO,
		MIF, HIF1A, SPRY-4,	CP, C-MET, CENP-C	IL-1β, TNF, IGFBP2,
		ALDOA,CD40,PSMB8,	CAST, F11R, FAS	FCER1G
		ERBA BETA,COL1A1,		
		BBC-1,FGF10, FBLN,		
		FAS, CPE, NOS2, CAST		
down-regulation	**50**	**150**	**132**	**42**
Annotated genes	SIN1,COL2A1,FN	COL2A1, TXN, TNCC2,	VDUP1, BACT2, TOM	COL1A1,PPP1R12A,
	HECTD1	OXT,TNC, TOM, HBB,	LDHA, PSMB7, G6PD,	SMCT1, IGF2, CD1D
		PTGS1, IRF2, PSMB7,	SMAD7, CD1D, SIN1,	SFN
		G6PD, CAT-1,CHID	HOXA7, HIF-1A	

Notes:Adult Injury(AI), Adult Control(AC), Neonate Injury(NI), Neonate Control(NC). Fold Change ≥ 2.0; *P* < 0.05.

Among the 825 differentially expressed genes in total, 62 corresponded to known genes with a unique identifier, and sourced from RefSeq and UniGene. The expression of annotated genes in each pair is shown in Table 3.

**Table 3:** Different expression of annotated genes between the every two groups

**Gene symbol**	**AI/AC**	**NI/NC**	**AC/NC**	**AI/NI**	**Gen bank accession**	**UniGene**
SMAD7	—	2.36^*^(0.025)	—	2.04^#^(0.040)	EE805013	Oar.1034
FCER1G	—	3.16^*^(0.037)	—	—	AJ318335	Oar.1043
CD1D	—	3.14^#^(0.018)	—	3.04^#^(0.047)	NM_001123001	Oar.1049
G6PD	—	—	2.75^#^(0.016)	3.70^#^(0.042)	NM_001093780	Oar.1073
EF-1	—	—	2.82^*^(0.036)	—	NM_001009449	Oar.1074
SIN1	3.25^#^(0.023)	—	—	2.24^#^(0.008)	NM_001009768	Oar.1093
VDUP1	—	—	—	2.46^#^(0.007)	EE783894	Oar.12992
OVAR	—	—	3.06^*^(0.019)	—	NM_001130934	Oar.13205
MMP7	—	—	—	2.60^*^(0.048)	NM_001136491	Oar.13267
COL1A1	—	2.87^#^(0.042)	5.90^#^(0.036)	—	DY492568	Oar.13279
LDHA	2.18^*^(0.030)	—	2.81^*^(0.030)	2.12^#^(0.026)	EE751721	Oar.13281
PRKAR1A	—	—	2.57^*^(0.039)	—	NM_001142517	Oar.13311
CAV1	—	—	4.25^*^(0.013)	—	DY493176	Oar.13316
F11R	—	—	—	3.56^*^(0.026)	DY502182	Oar.13343
HBB	—	—	5.21^#^(0.021)	—	DY522642	Oar.13537
SMCT1	—	3.18^#^(0.011)	—	—	EU048233	Oar.14460
PPP1R12A	—	2.42^#^(0.022)	—	—	EU370548	Oar.14621
IGFBP-2	——	12.98^*^(0.038)	—	—	NM_001009436	Oar.15563
HECTD1	2.79^#^(0.005)	—	—	—	EU370535	Oar.16241
PSMB7	—	—	2.46^#^(0.027)	4.04^#^(0.013)	EU366497	Oar.16276
COL2A1	5.10^#^(0.019)	—	3.74^#^(0.006)	—	ACJ06529.1	Oar.17681
IGF2	—	3.64^#^(0.042)	—	—	NM_001009311	Oar.376
IL-1β	5.57^*^(0.002)	5.55^*^(0.009)	—	—	DY502470	Oar.434
OXT	—	—	9.76^#^(0.050)	—	NM_001009801	Oar.444
PTGS1	—	—	3.67^#^(0.030)	—	NM_001009476	Oar.445
TNFα	4.03^*^(0.018)	3.52^*^(0.004)	—	—	DY503545	Oar.455
RAC1	—	—	—	2.09^*^(0.003)	EE785210	Oar.4580
NOD2	—	—	—	6.75^*^(0.046)	AM932877	Oar.4731
FZD3	—	—	—	4.05^*^(0.023)	DQ152955	Oar.4758
NFKBIA	—	—	3.08^*^(0.011)	4.15^*^(0.039)	EE815518	Oar.4761
MIF	—	—	2.16^*^(0.050)	—	NM_001078655	Oar.4767
SPRY-4	—	—	2.44^*^(0.040)	—	DQ152992	Oar.4778
TOM	—	14.37^#^(0.020)	—	14.13^#^(0.015)	NM_001035224	Oar.4810
TXN	—	—	2.89^#^(0.015)	1.94^#^(0.033)	NM_001009421	Oar.482
FN	3.38^#^(0.048)	—	4.65^*^(0.008)	—	FJ234417.1	Oar.4888
HOXA7	—	—	—	2.36^#^(0.009)	U61979	Oar.496
CAST	—	—	2.61^*^(0.032)	2.35^*^(0.015)	NM_001009788	Oar.498
ERBA BETA1	—	3.52^*^(0.004)	3.34^*^(0.010)	—	Z68307	Oar.500
ESR1	—	—	68.55^*^(0.000)	—	AY033393	Oar.505
TNC	4.82^*^(0.008)	—	4.56^#^(0.004)	—	DY475966	Oar.5104
TNCC2	—	—	3.30^#^(0.029)	—	NM_001112821	Oar.5156
TF	—	8.97^*^(0.023)	—	—	EE771342	Oar.552
CPE	—	—	3.66^*^(0.025)	—	AF063109	Oar.622
NOS2	—	—	2.28^*^(0.037)	—	AF223942	Oar.645
BCAT2	—	—	—	2.10^#^(0.025)	AF050173	Oar.655
HIF1A	—	—	2.31^*^(0.039)	2.35^#^(0.030)	EE755982	Oar.6671
FAS	—	—	3.58^*^(0.046)	7.19^*^(0.046)	NM_001123003	Oar.683
CP	—	—	—	7.91^*^(0.049)	NM_001009733	Oar.706
DCN	—	—	3.06^*^(0.044)	—	NM_001009218	Oar.718
ALDOA	—	—	2.27^*^(0.047)	—	EE814113	Oar.733
BBC1	—	—	2.03^*^(0.008)	—	EE773437	Oar.76
FGF10	—	—	5.15^*^(0.032)	—	NM_001009230	Oar.7650
PBR	—	—	5.35^*^(0.017)	—	NM_001009747	Oar.779
C-MET	—	—	—	6.06^*^(0.037)	NM_001111071	Oar.794
CAT-1	—	—	3.31^#^(0.026)	2.20^*^(0.041)	AF212146	Oar.798
SFN	—	2.45^#^(0.041)	—	—	NM_001009208	Oar.814
PSMB8	—	—	5.58^*^(0.036)	—	NM_001131030	Oar.8196
CENP-C	3.38^*^(0.048)	—	—	2.12^*^(0.028)	U35657	Oar.847
GRO	—	3.46^*^(0.042)	—	—	NM_001009358	Oar.963
IRF2	—	—	2.30^#^(0.027)	—	NM_001009740	Oar.966
CD40	—	—	8.45^*^(0.047)	—	EE821767	Oar.989
PPARG	—	3.72^*^(0.002)	—	—	NM_001100921	Oar.992

Notes:Adult Injury(AI), Adult Control(AC), Neonate Injury(NI), Neonate Control(NC).

*:up-regulation; #:down-regulation; ( ):P-value; —: no statistical significance (*P* > 0.05).

### Hierarchical clustering analysis

To investigate how gene expression varied across the samples, we performed hierarchical clustering analysis. In this analysis, samples were grouped according to their expression profile based on all genes, whether or not the genes were differentially expressed in the experimental (injured) versus the control (normal) group. A dendrogram shows the relationships among the expression levels of conditions. Our experiment consisted of 12 different conditions. The results of hierarchical clustering based on conditions showed a distinguishable gene expression profiling among samples (Figure 1D). Significant functional clusters included genes associated with wound healing, articular protection, repair integration, and energy metabolism. Such transcripts, including peroxisome proliferator activated receptor γ (PPARγ), trappin ovine molecule (TOM), mothers against DPP (Drosophila) human homolog 7 (SMAD7), nuclear factor-kappa B (NF-κB), hypoxia inducible factor-1α (HIF1-α), and lactate dehydrogenase (LDH) were regulated in their respective direction (up- or down-regulated) according to their change with tissue maturity/age and injury (Figure 3).

### Results by quantitative real-time RT-PCR

**Figure 4:**
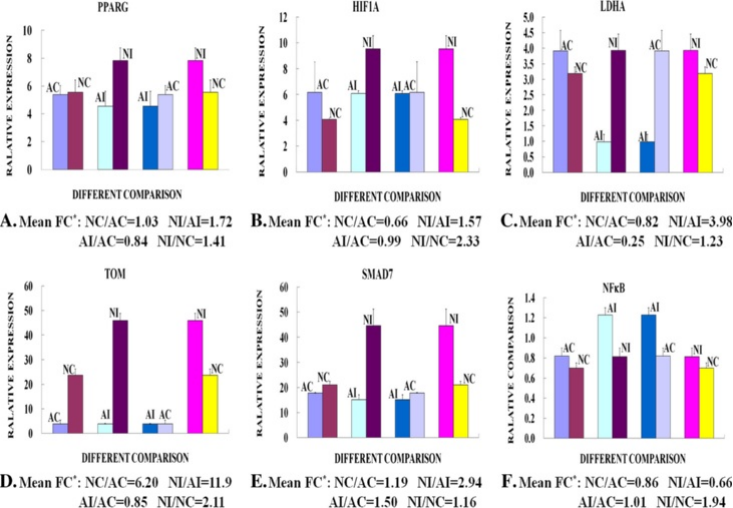
**Quantitative real-time PCR measurement of differential gene expression.** Adult injury (AI), adult control (AC), neonatal injury (NI), and neonatal control (NC). Quantification of transcript abundance indicates significant up-regulation of PPARγ (**A**), HIF1-A (**B**), LDH (**C**), TOM (**D**), SMAD7 (**E**), and NFκB (**F**) gene expression in neonatal injured articular cartilage compared with adult articular cartilage. The RT-qPCR data for all six target genes confirmed the results of microarray hybridization experiments. Mathematical means of expression are indicated below each age group, and mean fold differences for each target gene are also given numerically as ‘Mean FC’ under the abscissa. Two-sided t tests and an ANOVA were used for statistical analyses. ^*^P values < 0.05 were considered statistically significant.

Quantitative real-time RT-PCR was performed on the six up-regulated genes to validate the microarray results, including PPARγ, LDH, TOM, HIF1A, SMAD7, and NF-κB, which were associated with wound healing, articular protect, inflammation and energy metabolism according to literature [11-13]. We found a significant increase in mRNA abundance for PPARγ and TOM in neonatal injured articular cartilage (Figure 4). Fold change differences were similar or slightly greater than those measured by microarray profiles. In general, the quantitative real-time RT-PCR and microarray data agreed well for most samples, emphasizing the robustness of the microarray data.

## Discussion

Traumatic cartilage lesions represent a common symptomatic and disabling problem, which often requires surgical intervention to relieve pain and to prevent possible evolution towards secondary osteoarthritis [14]. In the present study, an ovine age-dependent *ex-vivo* articular cartilage model following acute injury was developed and characterized. Three pairs of adult and neonatal sheep articular cartilage were detected by cDNA microarray and validated by real time RT-PCR.

The repair of joint surface lesions largely depends on their size and depth [15,16], and the reproducibility of the injury is an important concern. With regard to the choice of the time course of post-injury, Lee et al. showed that the expression of specific catabolic and anabolic genes that regulate matrix remodeling and turnover after mechanical injury within 24 h is the most significant [17].

Differential gene expression in equine articular cartilage maturation was studied by Mienaltowski et al. [18]. However, the use of microarrays has not been reported in different developmental stages of ovine articular cartilage. In the present study, the up-regulation of collagen type II (COL2A1) and tenascin-C (TNC) was observed in neonatal articular cartilage, while transcripts encoding matrix proteins and growth factors were more abundant in adults, including collagen type I (COL1A1), decorin, and fibroblast growth factor 10. The current data are consistent with previous findings in horses and humans [18,19].

In adult injured articular cartilage versus normal articular cartilage, five annotated genes were significantly up-regulated. In contrast, the expression of four genes was slightly down-regulated. In particular, centromere protein-C, insulin growth factor binding protein 2, and LDH have not been previously linked to an imbalance of damage and repair in osteoarthritis, whereas, TNC and COL2A1 have already been reported [18].

Neonatal ovine lesional cartilage and normal articular cartilage were compared in this study. As expected, with the pattern of activation of inflammation and apoptosis-related genes broadly comparable to those reported in the adult [1], neonatal injured articular explants also had high levels of gene expression, such as interleukin 1β (IL-1β), tumor necrosis factor-α, growth-regulated oncogene α (GROα), and NF-κB.

In our study, transcripts encoding cartilage macromolecules and nuclear receptors, which play a role in cell-cell and cell-matrix interactions, tissue remodeling, and repair, were significantly more abundant in neonatal lesional articular cartilage compared with normal articular cartilage. There are two possible reasons for this finding. First, neonatal cartilage has different gene expression compared with adult cartilage, such as TOM, which may help its self-repair. Second, mechanical injury results in different responses between neonatal and adult cartilage. Our microarray analysis showed that transcripts, including PPARγ, HIF1-α, and SMAD7, are highly expressed in neonatal injured articular cartilage compared with the adult injury model.

PPARγ is expressed in chondrocytes and synoviocytes, and is present and functionally active in human chondrocytes [11]. Consistent with this finding, our study showed PPARγ was up-regulated 3.72-fold in injured neonatal articular cartilage compared with normal articular cartilage, whereas there was no significant difference in expression in the adult sheep injury model. Interestingly, there was also no difference in PPARγ expression in normal adult cartilage compared with neonatal cartilage. These findings suggested that neonatal cartilage showed a strong and unique response to mechanical injury. PPARγ has a significant protective effect and promotes cartilage repair in traumatized chondrocytes by several probable mechanisms. (1) Down-regulation of genes that encode catabolic factors could be involved in this process [20]. PPARγ agonists suppress the expression of inducible nitric oxide synthase and matrix metalloproteinase (MMP)-13 in human chondrocytes, as well as the expression of MMP-1 in human synovial fibroblasts. The inhibition of inducible nitric oxide synthase and MMP-13 induction is PPARγ dependent and occurs at the transcriptional level, probably through repression of NF-κB and AP-1 signaling [20]. The level of phosphorylation of JNK and p38 has also been shown to be diminished in response to specific stimuli in PPARγ-deficient mice [21]. (2) Anti-inflammatory effects are considered to mainly exert action through transrepressing proinflammatory genes in a DNA-binding-dependent manner [22,23]. Trauma can induce inflammatory responses, and also activate the expression of anti-inflammatory factors synchronously. PPARγ may be a potential therapeutic agent for treating articular cartilage injury and defects. Therefore, further study is required on how to enhance PPARγ expression to promote cartilage repair in adult injured articular cartilage.

To date, TOM is found in several tissues, including epithelia, lungs, and macrophages [12]. To the best of our knowledge, no report describing a protease inhibitor as a cartilage-sparing agent has been published. However, we detected TOM gene expression in ovine articular cartilage. TOM expression was significantly increased in neonatal ovine articular cartilage after acute mechanical injury, with a 14.1-fold increase compared with control adult tissue. However, there was no significant difference in TOM expression in the adult sheep injury model. Interestingly, TOM gene expression was increased 15.73-fold in normal neonatal articular cartilage compared with adult articular cartilage. TOM gene expression has inherently high levels in neonatal ovine articular cartilage, which is beneficial to cartilage repair. *In vitro* studies have shown that the immobilization of trappin-2/elafin extracellular matrix proteins in articular cartilage plays a protective role by preserving structural integrity of the tissue against damage caused by neutrophilic infiltration during inflammation [24]. Trappin-2 and elafin may promote cartilage repair through their anti-inflammatory activities, which appear to be independent of their anti-elastase activity [25]. All of these processes may be involved in the reason for a stronger repair capacity in neonatal articular cartilage than adult cartilage.

Articular cartilage following acute injury results in the activation of a series of signaling responses. In the present study, SMAD7 mRNA in chondrocytes was up-regulated by 2.36-fold in neonatal injured articular cartilage compared with normal articular cartilage. In contrast, SMAD7 was down-regulated 2.04-fold in adult injured articular cartilage compared with the neonate. There was no difference in SMAD7 expression between normal adult and neonatal cartilage. SMAD7 is involved in cell signaling, which is a transforming growth factor β (TGFβ) type I receptor antagonist. Overexpression of SMAD7 totally prevents TGFβ-induced proteoglycan synthesis in chondrocytes at the mRNA and protein level and completely antagonizes the effects of TGFβ on proliferation [26]. Therefore, SMAD7 may cause cartilage degeneration and accelerate the response of the injury by inhibiting TGFβ signaling. SMAD7 acts in a negative feedback loop to inhibit TGFβ activity because of its interaction with ligand-activated TGFβRI, and it interferes with the phosphorylation of receptor-associated Smads, preventing nuclear translocation of the activated Smad complexes [27]. The effects of IL-1β on SMAD7 expression in human articular chondrocytes are mediated through the NF-κB pathway [13]. Interestingly, SMAD7 has been reported to regulate the NF-κB pathway. SMAD7 is able to block the TGFβ-induced phosphorylation of IκB, resulting in a decrease in NF-κB DNA binding [28]. Other studies have indicated that SMAD7 can also act as an NF-κB activator in some conditions [29]. In addition, a recent study showed that SMAD7 overexpression in transgenic mouse epidermis at levels comparable to those seen in pathological states is insufficient to block TGFβ or bone morphogenetic protein signaling, but instead produces striking phenotypes due to degradation of β-catenin through a novel mechanism involving Smad7 and Smurf2 [30].

SMAD7, NF-κB, and TGFβ pathways play a vital role in articular cartilage development and homeostasis. Therefore, a potential new mechanism for pathway cross-talk has important implications for the understanding of maturation and repair of articular cartilage.

## Conclusions

There are significant differences in gene expression between neonatal and adult ovine articular cartilage following acute injury. These differences are partly due to intrinsic differences in the process of development and partly to different biological responses to mechanical trauma between neonatal and adult articular cartilage. Of these, PPARγ and TOM could be novel target molecules and potential chondroprotective agents involved in cartilage injury and complete repair.

## Abbreviations

PPARγ: Peroxisome Proliferator Activated Receptor γ; TOM: Thappin Ovine Molecule; SMAD7: Mothers Against Dpp (Drosophila) Human Homolog 7; NF-κB: Nuclear Factor-Kappa B; HIF1-α: Hypoxia Inducible Factor 1α; LDH: Lactate Dehydrogenase; GAPDH: Glyceraldehyde-3-Phosphate Dehydrogenase; COL2A1: Collagen Type Ii; TNC: Tenascin-C; COL1A1: Collagen Type I; IL-1β: Interleukin 1β; GROα: Growth-Regulated Oncogene α; MSCs: Mesenchymal Stem Cells; MMP-13: Matrix Metalloproteinase-13; TGFβ: Transforming Growth Factor β.

## Competing interests

The authors declare that they have no conflict of interests related to this work.

## Authors’ contributions

HW had full access to all of the data in the study and takes responsibility for the integrity of the data and the accuracy of the data analysis. Conception and design: XX, HW, QZ. Analysis and interpretation of the data: XX, HW. Drafting of the article: XX, QZ, HW. Critical revision of the article for important intellectual content: XX, QZ, HW. Final approval of the article: XX, QZ, HW, LZ, PL. Statistical analysis: XX, HW, QZ. Administrative, technical, or logistic support: HW,QZ, LZ, PL. Collection and assembly of data Acquisition of data: XX, HW, LZ, PL. All authors read and approved the final manuscript
